# A new nasal cavity nursing methods application in patients with mechanical ventilation

**DOI:** 10.12669/pjms.294.3636

**Published:** 2013

**Authors:** Liuqing Wei, Gang Qin, Xining Yang, Meichun Hu, Fufu Jiang, Tianwei Lai

**Affiliations:** 1 **Liuqing Wei, Department of Intensive Care Unit, Guangxi Hospital for Nationalities, Nanning, Guangxi, 530001, China.**; 2 **Gang Qin, Department of Intensive Care Unit, Guangxi Hospital for Nationalities, Nanning, Guangxi, 530001, China.**; 3 **Xining Yang, Department of Intensive Care Unit, Guangxi Hospital for Nationalities, Nanning, Guangxi, 530001, China.**; 4 **Meichun Hu, Department of Intensive Care Unit, Guangxi Hospital for Nationalities, Nanning, Guangxi, 530001, China.**; 5 **Fufu Jiang, Department of Intensive Care Unit, Guangxi Hospital for Nationalities, Nanning, Guangxi, 530001, China.**; 6 **Tianwei Lai, Department of Intensive Care Unit, Guangxi Hospital for Nationalities, Nanning, Guangxi, 530001, China.**

**Keywords:** Atomizing nasal cleaning method, Mechanical ventilation, Ventilator- associated pneumonia

## Abstract

***Objective:*** To compare different nasal cavity nursing methods on mechanically ventilated patients.

***Methods:*** According to acute physiology and chronic health evaluation (APACHEII), 615 cases of mechanically ventilated patients were divided into group A, group B and group C by stratified random method. Traditional oral nursing plus aspirating secretions from oral cavity and nasal cavity q6h were done in group A. Based on methods in group A, normal saline was used for cleaning nasal cavity in group B. Besides the methods in group A, atomizing nasal cleansing a6h was also used in group C. Incidence rate of Ventilator-Associated Pneumonia (VAP) and APACHE II scores after administrating were compared. The correlation between APACHE II score and outcomes was analyzed by Spearman-rank correlation.

***Results:*** In group A, incidence of VAP was 36.76%, group B was 30.24%, group C was 20.38%, and the difference was statistically significant. APACHE II scores in group C were significantly lower compared with group A and B. APACHE II score was negatively correlated with clinical outcomes.

***Conclusions:*** For mechanically ventilated patients, nasal nursing can’t be ignored and the new atomizing nasal cleaning is an effective method for VAP prevention.

## INTRODUCTION

Ventilator-associated pneumonia (VAP) is the most common type of nosocomial pneumonia, as well as one common complication and cause of death of patients with mechanical ventilation in intensive care medicine (ICU).^1^^,^^2^ The established artificial airway damaged the normal respiratory anatomy function, resulting in a high incidence of VAP in mechanical ventilated patients.^3^^-^^6^ VAP is related with many factors, including original diseases and invasive medical procedures. The original disease is one of the most important personal reasons, and the invasive medical procedure is one of the most important external factors. Studies showed that^6^ nursing staff plays an important role in the prevention of VAP. With nursing and prevention measures being further improved, the incidence of infection decreased year by year. From January 2010 to August 2012, 615 mechanically ventilated patients received 3 different respiratory tracts nursing in our hospital, each nursing effect on VAP prevention was studied as follows.

## METHODS

***Subjects:*** From January 2010 to August 2012, 615 (including 347 males and 268 females) mechanically ventilated patients received 3 different respiratory tract nursing in our hospital. Age ranged from 21 to 92, with a mean age of 61.3 years old. Among these patients, 94 cases suffered from cerebral hemorrhage, 62 cases had type 2 diabetes, 54 cases had myocardial infarction, 78 cases suffered from large operation, 43 cases had acute respiratory distress syndrome, 35 cases had multiple organ function failure, 31 cases suffered from disseminated intravascular coagulation, 29 cases suffered from other diseases.

***Inclusion criteria:*** One patient with indwelling time > 24h; 2 collect deep sputum for culturing before intubation; 3 time for Ventilator from 2 to 65 Days, the average time is 16.4 days. The patients who were on ventilation for more than 65 days were dropped from this study.

***Exclusion criteria:*** patients who used intubation in the past 30 days.

Manners of Endotracheal Intubation and Time of Ventilation: Three manners of endotracheal intubation were employed, including tracheostomy, nasal endotracheal intubation and oral endotracheal intubation. Ventilators would assist breath after endotracheal intubation. These patients including 28 cases of tracheotomy, 193 cases through mouth, 394 cases through nose. Four hundred and seventy-five patients’ continuous ventilation time less than 15d, 53 patients less than 30 day, 42 patients less than 45 day, 18 patients less than 60 day, 6 patients more than 61 day.

***Diagnosis Criteria:*** We diagnosed the disease according to the “criteria for the diagnosis and treatment guideline of hospital-acquired pneumonia”.^7^ We judged them by which pulmonary infection occurred 48h after artificial ventilation, or the cultivated bacterial strains were different from intubation before, referring to clinical manifestation, radiological examination and etiological examination.

Specimen selection: Specimens were collected from mechanical ventilation works before and after 48h on 3 consecutive days, respectively. Then continuous sampled each time every 48 hours, until 48h after extubation. The sputum was collected from lower respiratory tract secretions using sterile sputum collector.

Bacterium culture and identification: Smear and quantitative bacteria culture were done immediately after specimens were received. The count of bacteria > 105CFU/ml or fungus ≥ 104CFU/ml were regarded as pathogenic bacteria. If the same type of bacteria was detected continuously from a patient, it would be identified as one strain.

***Trial Grouping:*** According to the score (APACHE II) and the stratified random method, 204 cases were divided into group A, 205 cases into group B, 206 cases into group C. There were no significant differences among the sex, occupation, disease, and intubation manners.

***Intervention strategy:*** 1 Group C, nasal cavity cleaning by spray. The operation was as follows: oral care, suctioned from catheter, suctioned from oral cavity, suctioned from nasal cavity, spraying (nozzle along the nasogastric tubes and tracheal catheters that aimed at nasal wall but did not enter into nose) when head toward back slightly, 4 sprays per nostril each time, 6 hours a round. In order to improve executive power, every nurse should learn to do these. All the data were written down by appointed persons. 2 Group B, the operations were as follows: oral care, suctioned from catheter, suctioned from oral cavity, cleaned nasal vestibule by saline cotton swab. 3 Group A, the operations were as follows: oral care, suctioned from catheter, suctioned from oral cavity, suction from nasal cavity.

***Atomizing nasal cleaning:*** In group A, only suctioning, and without cleaning nasal cavity, which leaded to bacteria also surviving on the surface of mucus. In group B, it was not easy to clean the nasal cavity thoroughly that only used saline cotton swabs to clean nasal vestibule after suctioning the secretions. The main reason might be that tracheal catheters hinder us to clean the dead corner of deep part of nasopharynx easily, which caused the secretion accumulated there. In group C, spraying with Physiological Seawater Nasal Spray nozzle along the nasogastric tubes and tracheal catheters that could cleaned the deep part of nasopharynx and reduced the incidence of VAP caused by colonized bacteria in nasopharynx. The ingredient of Physiological Seawater Nasal Spray is sea salt, and its concentration equal to the concentration of body fluid. The Physiological Seawater Nasal Spray is rich in minerals from sea, such as silver and zinic with bactericidal or antiviral actions, cooper with diminishing inflammation function, manganese with anti-anaphylaxis. Physiological Seawater Nasal Spray could also moistened nasal cavity, dissolve the secretion, get rid of pathogenic microorganism in the mucus, and reduce the incidence of VAP which caused by bacteria accumulation in nasopharynx and on the external wall of catheter. This manner is simple and has a high compliance.

***Observing indexes:*** Participant: (1) VAP occurred when 48h after mechanical ventilation or artificial tracheas were removed after mechanical ventilation. Two patients who suffered from pneumonia before, while new pathogenic bacterias were also detected now. Observing indexs: 1temperature ≥ 38.0ºC; (2) purulent secretion was suctioned from airway; (3) new pathogenic bacterias were detected from patients who had suffered from infection or pathogenic bacterias; (4) bacterias were detected from patients who had not suffered from infection; (5) X ray detection taken at bedside showed a new shaded area or a enlarged shaded area in lung; 6 leukocyte count > 10.0×109/L or <4.0×109/L, with or without nuclear shift to the left.

***Statistical analysis:*** Statistical analysis was performed by using SPSS 10.0. ÷2 test or Fisher’s exact test were used in enumeration data, and Spearman rank correlation was used in correlation analysis.

## RESULTS

***Incidence of VAP: ***The incidence of VAP was classified into 3 sections, including the total incidence, the early incidence (VAP occurred in 4 days when mechanical ventilation was used), the delayed incidence. The total incidence of group A was 36.76% (75 cases in 204 patients), group B was 30.24% (62 cases in 205 patients), group C was 20.38% (42 cases in 206 patients). From the Table-I, shows that there were significant differences among the three groups (p < 0.05). The total incidence in group C was lower significantly compared with the group A and group B (both p<0.01), and group C was lower significantly compared with the group B (p<0.05). In addition, the early incidence and the deferred incidence of group A were also significantly higher compared with that of group B and group C (p<0.05 and p<0.01, respectively). Meanwhile, the early incidence and deferred incidence of group C were significantly lower compared with that of group B (p<0.01 and p<0.05, respectively) (Table-I).

***Sputum culture analysis:*** 179 sputum specimens were cultured, including 75 specimens in group A, 62 specimens in group B and 42 specimens in group C. In group A, 215 strains of bacteria were identified, including 104 strains of gram positive bacteria, 104 strains of gram negative bacteria and 15 strains of fungus. In group B, 189 strains of pathogenic bacterium were isolated, including 89 strains of gram positive bacteria, 85 strains of gram negative bacteria and 15 strains of fungus; In group C, 78 strains of pathogenic bacteria were isolated, including 35 strains of gram positive bacterium, 36 strains of gram negative bacterium and 7 strains of fungus. As shown in Table-II, we discovered that for all of the index (infected cases, pathogenic bacterium, gram-positive bacteria, gram-negative bacteria and fungus), group C was significantly fewer compared with those of group A and B (all p<0.01, except for p<0.05 in fungus) and group B was also significantly lower compared with those of group A (all p<0.01, but not difference in fungus). In addition, we also found that Bauman Acinetobacter, Pseudomonas aeruginosa, Klebsiella, E. coli were the main bacteria in this three group (data not shown).

**Fig.1:**
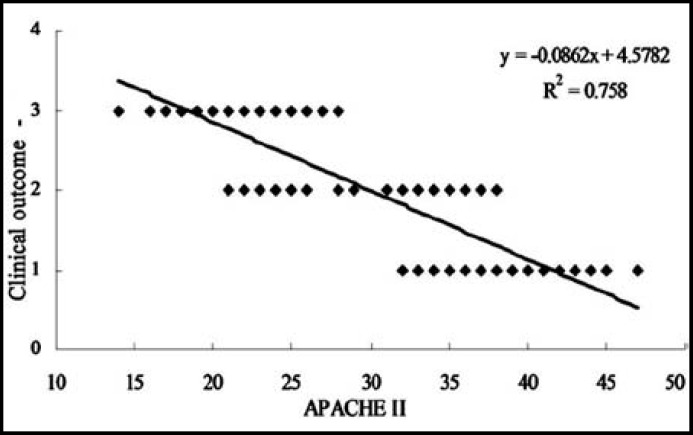
Comparison of APACHE II score among three groups. **P<0.01, C group vs A group; #P<0.05, C group vs B group; $P<0.05, B group vs A group

***APACHE II score:*** In this study, APACHE II score was also observed after administrating the three groups nursing methods. The results indicated that after the nursing assistants, the APACHE II scores in group C were significantly lower compared with that in group A and group B (Fig.1). (p<0.01 and 0.05, respectively). Moreover, the APACHE II scores in group B were also significantly lower compared with that in group A (P<0.05).

**Fig.2:**
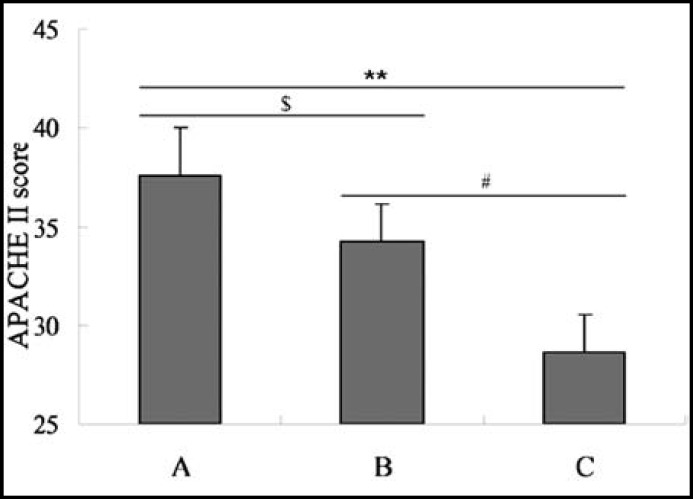
Correlation analysis between APACHE II score and clinical outcome

Correlation between the APACHE II score and the prognosis: In order to assess the relationship between the APACHE II score and the disease prognosis, the correlation analysis was performed. From Fig.2, we could find that the APACHE II score was negatively correlated with the clinical outcome (r=-0.87063, P<0.05).

**Table-I:** VAP rate (%) in the three groups. Mention p value here in this table

Group	Total Cases	Total Vap Cases (%)	Early Vap Cases (%)	Delayed Vap Cases (%)
A	204	75(36.76)	32(15.7)	54(26.5)
B	205	62(30.24)$	24(11.7) $	46(22.4) $
C	206	42(20.38)**, ##	6(2.9) **, ##	29(14.0) **, #

**P<0.01 represents the group C VS group A; ##P<0.01 represents the group C VS group B; $P<0.05 represents the group B VS group A

**Table-II:** Sputum culture results of three Groups.

Group	Infected cases (%)	Pathogenic bacterium (n)	Gram-positive bacterium (n)	Gram-negative bacterium (n)	Fungus (n)
A	75(36.76)	215	104	96	15
B	62(30.24)$$	189$	89$	85$	15
C	42(20.39)**,##	78**,##	35**,##	36**,##	7*,#

**P<0.01, *P<0.05 represents the group C VS group A; ##P<0.01, #P<0.05 represents the group C VS group B; $P<0.05 represents the group B VS group A.

## DISCUSSION

Large amounts of dirt and germs will accumulate in the nasal cavity under normal situation. Normally, the receptors which could bind to bacteria distributed on the surface of nasopharyngeal epithelial cell are covered by nectin of cellulose.^8^ The nectin could ensure the bacteria can’t colonize at the epithelial cells.^9^ There was stress among the serious patients who needed to spile, such as hypotension, hypoxia, acidosis, and act. At the time, proteolytic enzymes of secretion in pharyngeal were elevated, which could catalyze the nectin of cellulose that on the surface of nasopharyngeal epithelial cell. 

Finally, receptors on the surface of cells were exposed, and the opportunity of colonization was increased. At the same time, nose failed to clean itself, which lead to bad situation in nasal cavity. So bacteria accumulated and multiplied faster. For the tracheal catheter hindering swallow, secretion accumulated in nasal cavity and oral cavity most of the time. Especially in coma patients, bacteria in the secretion along the catheter entry into respiratory tract through sub-glottic area, which leaded to bacteria move down and increased the incidences of VAP.^10^ Routine respiratory tract management might often ignore the complete cleaning of the secretion in nasal cavity, especially in patients who needed indwelling gastric tubes or indwelling tracheal catheters. The patients were easy to have complications by nasosinusitis, because it was difficult to remove the secretion.^6^ So clearing the contaminated nasal cavity immediately, especially in the deep part of the cavity, were very important for VAP prevention. Keeping clean of the nasal cavity, and getting rid of feculence stayed in the nasal cavity or on the wall of the tracheal catheters, were key factors to reduce the incidence of VAP.

In the present study, the incidence of VAP in group C was lower than that in group B (p < 0.05) and group A (p < 0.01). Because of swallowing reflex, cough reflex and lower respiratory tract ciliary movement weakened or lost in patients with mechanical ventilation, the throat secretions and colonized bacteria were accumulated on the external wall of catheter. So the accumulated bacteria could form “mucus paste” and became warehouse of bacteria. Cleanliness of nasal cavity and nasopharynx were highly associated with the infection of lower respiratory tract.

This study showed that the incidence of VAP in group C was 20.38%, which was lower than group B (30.24%) and group A (36.76%). The results demonstrated that cleaned nasal vestibule by saline cotton swabs could not clean the bacteria stayed in the deep part. Moreover, normal saline have the capacity of sterelization and antivirus, so method of B can also prevent VAP. There was a tight relationship between VAP and nasal cavity nursing, so searching for an effective nasal cavity nursing was very important.

The main microorganisms of this study were Gram-negative bacteria, including Bauman Acinetobacter and Pseudomonas aeruginosa. In addition, there were also a few Gram-positive bacteria and fungus. Within 48 hours of mechanical ventilation, normal Gram-positive streptococci changed into Gram-positive bacteria with powerful pathogenicity. When these Gram-positive bacteria inspirated into lungs, which became the main reasons that caused VAP on patients with mechanical ventilation.

The APACHE II scores were also assessed after the nursing methods administrating which found that group A showed a significant lower APACHE II score compared with the group B and C. So the results indicated that the new atomizing nasal cleaning method could improve the APACHE II score significantly, which was consistent with the previous study.^6^ The correlation analysis indicated that there was a negative correlation between the APACHE II score and the disease outcomes, which was consistent with the previous studies.^11^,^12^ A better outcome would be received when the APACHE II scores decline, so the group A could improve the disease prognosis with a good outcome.

To summarize, nasal cavity nursing is important for the patients with catheters, which is consistent with the earlier studies.^13^,^14^ The improvement of the nasal cavity method and strengthening of nasal cavity nursing could reduce VAP caused by colonized bacteria in nasopharynx and oral cavity. Prevention and nursing of VAP is a systemic engineering, plays a key role in improving medical quality. To prevent the colonization of pathogenic bacteria in nasopharynx, the management of respiratory tract is very important. In addition, changing position, effective aspirating secretion, strict sterilization, and effective oral nursing could stop the moving down of bacteria in throat, which is an important factor for VAP prevention. Thus, the new nasal spray cleaning method could reduce length of stay, the average hospitalization expenses, mortality and improve the medical and nursing quality.

## Authors Contributions:

***LW:*** Performed the experiment and prepared the original manuscript.

***GQ: ***Performed the experiment and reviewed the manuscript for publication.

***MH:*** Contributed in data collection, statistical analysis and manuscript writing.

***XY:*** Was involved in clinical management of patients,

***TL:*** Prepared the different nasal cavity nursing methods. 

***FJ:*** Designed the protocol and critically reviewed the manuscript for final publication.

## Notes

***Declaration:*** We declare that we have no conflict of interest.
